# A Personalized Arrhythmia Monitoring Platform

**DOI:** 10.1038/s41598-018-29690-2

**Published:** 2018-07-30

**Authors:** Sandeep Raj, Kailash Chandra Ray

**Affiliations:** Department of Electrical Engineering, Indian Institute of Technology Patna, Bihta, 801103 India

## Abstract

Arrhythmia detection is the core of cardiovascular disease diagnosis. Though, there is no such generic solution for detecting the arrhythmias at the moment they occur which is due to the non-stationary nature and inter-patient variations of ECG signals. The feature extraction and classification techniques are significant tools widely used in the automated classification of arrhythmias. This study aims to develop a personalized arrhythmia monitoring platform allowing real-time detection of arrhythmias from the subject’s electrocardiogram (ECG) signal for point-of-care usage. A novel method, i.e. discrete orthogonal stockwell transform (DOST) technique for feature extraction is employed to capture the significant time-frequency coefficients to constitute the feature set representing each of the ECG signals. These coefficients or features are classified using artificial bee colony (ABC) optimized twin least-square support vector machine (LSTSVM) for classifying the different categories of ECG signals. The ABC optimizes the dimension of the feature set and the learning parameters of the classifier. The proposed method is prototyped on the commercially available ARM-based embedded platform and validated on the benchmark MIT-BIH arrhythmia database. Further, the prototype is evaluated under two schemes, i.e. class and personalized schemes which reported a higher overall accuracy of 96.29% and 96.08% in the respective schemes than the existing works to the state-of-art CVDs diagnosis.

## Introduction

The world health organization places the cardiovascular diseases (CVDs) as the leading cause of deaths globally. These CVDs occur due to the long-term effect of cardiac arrhythmias. Generally, the cardiac arrhythmias are not often life-threatening but can lead to cardiac death or heart failure in long run and required to be detected on time. Computerized electrocardiography (ECG) is a widely used diagnostic measure to interpret the function of subject’s heart. The automatic monitoring of heartbeats is divided into four stages (i) pre-processing (ii) R-peak detection and ECG signal segmentation (iii) feature extraction and classification. The initial two stages i.e. preprocessing and R-peak detection and heartbeat segmentation are widely explored in the literature. However, the improvements can be done in the feature extraction and classification stages. Several methods have been throughly studied in the domain of efficient and automatic monitoring of heartbeats. The detection and recognition of ECG signals mainly consist of the combination of an efficient feature extraction technique followed by the machine learning algorithms^1–13^. In the feature extraction stage, time domain^7,14^, frequency domain^8^, time-frequency domain^9,10^, and statistical techniques^15–17^ are commonly used to capture the significant features of the heartbeats. However, each of these techniques exhibit certain limitations in their domain of analysis. For a time-domain input signal, the classical Fourier transform (FT) fails to provide any information regarding the time of occurrence of the frequency components. The ambiguity of FT is overcome by short-time Fourier transform (STFT), however it is limited to stationary signals only due to the constant window length. The shortcoming of STFT is overcome by Wavelet transform (WT), however the choice of mother wavelet and the levels of decomposition of an input signal remains a challenge. The S-transform overcome the challenge in WT, however it provides a redundant representation of the time–frequency plane and hence, is computationally expensive. Therefore, an efficient version of ST i.e. DOST is employed in this study to extract significant characteristics from the ECG signals. These feature extraction methods are combined with the classification tools which include the artificial neural network (ANN)^11,18^, support vector machines (SVMs)^10^, k-nearest neighbor and many more. However, these techniques fail to provide a generic solution and suffer from the drawbacks such as (1) a general purpose modeling is infeasible due to high inter-subject variation of heartbeats (2) due to the non-stationary nature of ECG, the QRS complex, P waves, and RR-intervals vary from one signal to another depending upon the lifestyle of subject^3^ and (3) the discriminative capability of the extracted features vary significantly for different heartbeat patterns (4) the training and testing dataset consists of heartbeats from the same patient which is not feasible for practical applications.

This paper addresses the aforementioned issues by presenting a novel analysis method i.e. discrete orthogonal stockwell transform (DOST) based artificial bee colony (ABC) optimized least-square twin support vector machines (LSTSVMs) to detect and classify different categories of ECG signals. The DOST localizes the spectrum and retains the phase properties by extracting the time-frequency features from an input ECG signal. The artificial bee colony (ABC) technique gradually optimizes the learning parameters for the LSTSVM classifier to develop an efficient model for the extracted features. The proposed method is implemented on the commercially available ARM-based embedded platform and validated on the benchmark MIT-BIH arrhythmia database^19^. The developed platform is evaluated under two assessment schemes i.e. class and personalized schemes. The personalized scheme is suitable for practical applications, since the training and testing datasets comprises of records of different patients (i.e. considering the inter-individual variability in the data). The prototype is trained in off-line mode while the results are reported on the testing dataset. More particularly, this study proposes the validation of developed platform on the MIT-BIH data for real-time verification of the proposed method allowing monitoring of arrhythmias for point-of-care applications.

## Results

### Database Setup

The proposed method is validated over the benchmark MIT-BIH arrhythmia database^19^ and evaluated under two analysis schemes i.e. setup I (class-oriented) and setup II (personalized) schemes respectively.

***Setup I*** (Class-oriented scheme): In this scheme, all the 48 records of the database comprising beat 110109 labels are utilized for analysis. The experiments are carried out on the training and testing datasets constituted by randomly selecting a fraction of ECG signals from each class and further divided into sixteen clusters where each class of ECG signal represents each cluster. Here, each class of heartbeat is represented using each cluster. The training dataset comprises of 23996 signals, (i.e. 21.26%) while rest of heartbeats are utilized for the testing purpose. Table 1 presents the training and testing datasets utilized for the experimental purpose. Further, the optimal parameters for the classifier are determined by performing 14 fold cross-validation on the training dataset, while the testing is performed on the testing dataset^7^. The final accuracy is computed by averaging the accuracy of all the folds.

**Table 1:** Summary of datasets in category based analysis scheme.

ECG signal Type - Annotation	Total	Training	${{\bf{T}}}_{{\bf{R}}}$(%)	Testing
Normal (NOR) - N	75017	11253	23	63764
Left Bundle Branch Block (LBBB) - L	8072	2825	35	5247
Right Bundle Branch Block (RBBB) - R	7255	2539	35	4716
Atrial Premature Contraction (APC) - A	2546	891	35	1655
Preventricular Contraction (PVC) - V	7129	2495	35	4634
Paced Beat (PACE) - P	7024	2458	35	4566
Aberrated Atrial Premature Beat (AP) - a	150	75	50	75
Ventricular Flutter (VF) - !	472	236	50	236
Fusion of Ventricular and Normal Beat (VFN) - F	802	401	50	401
Blocked Atrial Premature Beat (BAP) - x	193	97	50	96
Nodal (Junctional Escape Beat) - j	229	115	50	114
Fusion of Paced and Normal Beat (FPN) - f	982	491	50	491
Ventricular Escape Beat (VE) - E	106	53	50	53
Nodal (Junctional) Premature Beat (NP) - J	83	42	50	41
Atrial Escape Beat (AE) - e	16	8	50	8
Unclassificable Beat (UN) - Q	33	17	50	16
Total	110109	23996	21.79	86113

***Setup II*** (Personalized scheme): In this scheme, the AAMI standard^14,20–22^ is followed where four records i.e. 102, 104, 107, and 217 are excluded from the datasets, i.e. the analysis is carried out on the remaining 44 records. The 16 classes of ECG signals from the MIT-BIH arrhythmia database are mapped into five bigger classes namely N (beats originating in the sinus mode), S (supraventricular ectopic beats (SVEBs)), V (ventricular ectopic beats (VEBs)), F (fusion beats), and Q (unclassifiable beats). Under this scheme, two experiments are performed following Ince *et al*.^21^ (Setup IIA) and Chazal *et al*.^7^ (Setup IIB). In the setup IIA, the first 20 records (within a range of 100–124), which include representative samples of routine clinical recordings, are used to select representative beats to be included in the common training data. The rest 24 recordings (within a range of 200–234) contain ventricular, junctional, and supraventricular arrhythmias. A total of 83648 beats from all 44 records are used as test patterns for performance evaluation. a stopping criterion is decided which is the minimum train classification error level that is set to 1% to prevent over-fitting. In setup IIB, the training and testing datasets are constituted by equally splitting the records i.e. each dataset comprises of 22 records. Further, the optimal parameters for the classifier are determined by performing 22 fold cross-validation on the training dataset, while the testing is performed on the testing dataset^7^. The final accuracy is computed by averaging the accuracy of all the folds. Both these experiments are performed to have a fair comparison among the existing methods in the literature.

### Proposed Method Flow

**Figure 1:**
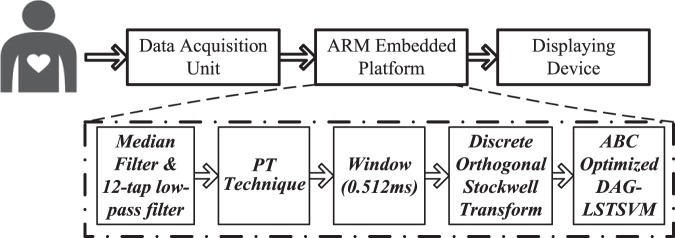
Proposed method flow.

The block diagram of the proposed method flow is depicted in Fig. 1.

- **Data Acquisition Unit:** The real-time input ECG signals are generated using MIT-BIH arrhythmia data and provided as input to the embedded platform for processing and analysis. Since, the monitoring platform is trained in off-line mode, therefore ECG signal from the testing datasets only for both the schemes i.e. setup I and II are generated.
- **ARM based embedded platform:** The proposed method is implemented on the ARM embedded platform to provide real-time monitoring of electrocardiogram signals. The real-time input ECG signals are input of the ARM based embedded platform. The median filters and low-pass filter to remove the baseline wander and high-frequency noise from the corresponding ECG signals. Thenafter, the R-peak in the ECG signal is detected within the input ECG signals. The discrete orthogonal stockwell transform features (i.e. 1 × 256) are computed to represent the input ECG signals. The selection of features and tuning of the least square twin SVM classifier is done using the artificial bee colony (ABC) method. Once the feature reaches the classification stage, the computation gets started and the input signal is assigned to a particular class.
- **Displaying Device:** The output class detected by the embedded platform is displayed on the 16 × 2 liquid crystal display (LCD).

### Performance metric evaluation

The classification performance for each class of heartbeat is computed using five standard metric parameters i.e. sensitivity (*S*_*E*_), positive predictivity (*P*_*P*_), accuracy (*A*_*C*_), error rate (*E*_*R*_) and *F*-score (*F*_*s*_). The sensitivity is the rate of correctly classified events among all of the events, *S*_*E*_ = *TP*/(*TP* + *FN*). The *P*_*P*_ is the rate of correctly classified events in all of the detected events, *P*_*P*_ = *TP*/(*TP* + *FP*). The *A*_*C*_ is the ratio of the number of correctly classified patterns to the total number of patterns classified, *A*_*C*_ = (*TP* + *TN*)/(*TP* + *TN* + *FP* + *FN*) and F-score (*F*_*s*_) is defined as (2*TP*/2*TP* + *FN* + *FP*). These three aforesaid parameters are computed for each category of ECG signal over the MIT-BIH database.

The class of heartbeats detected by the developed platform for each tested signal is compared with the annotations file to formulate the results and reported in the confusion matrix as in Tables 2, 3 and 4 for setup I, IIA and IIB i.e. class-oriented and personalized assessment schemes respectively. This matrix maps the correctly classified and misclassified signals into their subsequent classes. In the confusion matrix, the column represents the predicted signals detected by the platform while the row represents the ground truth (i.e. labels provided in the database). In Table 2, among the 86113 testing signals, 82918 signals are correctly detected achieving a high accuracy of 96.14% with an error rate of 3.86%. In the personalized scheme (Setup IIA), an accuracy of 96.08% is achieved i.e. out of 83648 signals 80379 signals are correctly detected by the prototype summarized in Table 3. While in setup IIB as in Table 4, the platform reported an accuracy and error rate of 86.89% and 13.11% respectively i.e. out of 49711 signals, 43194 signals are correctly detected. The accuracy reported for the classes ‘e’ and ‘Q’ as in Tables 2, 3 and 4 is very low i.e. which is due to the less amount of data considered for training purpose.

**Table 2:** Confusion matrix and performance metrics in setup I (class scheme).

	Predicted Labels																	Total	*S*_*E*_ (%)	*P*_*P*_ (%)	*F*_*S*_ (%)
		N	L	R	A	V	P	a	!	F	x	j	f	E	J	e	Q				
Ground Truth	N	61910	130	0	1193	311	0	61	23	57	0	33	27	0	0	11	8	63764	97.09	99.11	98.09
	L	122	5051	0	0	74	0	0	0	0	0	0	0	0	0	0	0	5247	96.26	94.02	95.13
	R	157	0	4461	71	19	0	0	0	0	0	0	0	0	8	0	0	4716	94.59	100	97.22
	A	32	23	0	1487	14	0	0	4	95	0	0	0	0	0	0	0	1655	89.85	53.78	67.28
	V	86	15	0	0	4369	0	0	79	71	0	0	14	0	0	0	0	4634	94.28	90.59	92.4
	P	23	109	0	0	0	4357	0	0	0	0	0	77	0	0	0	0	4566	95.42	99.89	97.6
	a	13	0	0	7	3	0	52	0	0	0	0	0	0	0	0	0	75	69.33	46.02	55.32
	!	14	32	0	0	19	0	0	171	0	0	0	0	0	0	0	0	236	72.46	60.64	66.02
	F	41	0	0	0	11	0	0	0	349	0	0	0	0	0	0	0	401	87.03	61.01	71.74
	x	11	0	0	3	0	0	0	5	0	77	0	0	0	0	0	0	96	80.2	100	88.34
	j	14	0	0	0	0	0	0	0	0	0	96	0	0	4	0	0	114	84.21	74.42	79.01
	f	23	11	0	0	0	4	0	0	0	0	0	453	0	0	0	0	491	92.26	78.92	85.07
	E	8	0	0	0	0	0	0	0	0	0	0	0	45	0	0	0	53	84.91	100	91.84
	J	3	0	0	4	2	0	0	0	0	0	0	0	0	32	0	0	41	78.05	72.73	75.29
	e	4	1	0	0	0	1	0	0	0	0	0	0	0	0	2	0	8	25	15.38	19.05
	Q	6	0	0	0	1	0	0	0	0	0	0	3	0	0	0	6	16	37.5	42.86	37.5
	Total	62467	5372	4461	2765	4823	4362	113	282	572	77	129	574	45	44	13	14	86113	96.29	96.29	76.06

**Table 3:** Confusion matrix and performance metrics in setup IIA (personalized scheme) based on datasets in^25^.

Predicted Labels													
		N	S	V	F	Q	Total	TP	FN	FP	*S*_*E*_ (%)	*P*_*P*_ (%)	*F*_*S*_ (%)
Ground Truth	N	73242 (40648)	863 (707)	439 (342)	77 (48)	29 (21)	74650 (41766)	73242 (40648)	1408 (1118)	1207 (1186)	98.11 (97.32)	98.38 (97.16)	98.24 (97.24)
	S	649 (661)	1614 (1461)	191 (189)	7 (4)	9 (5)	2470 (2320)	1614 (1461)	856 (859)	1178 (1009)	65.34 (62.97)	57.80 (59.14)	61.35 (61.00)
	V	395 (353)	287 (281)	5128 (4083)	63 (72)	26 (31)	5899 (4820)	5128 (4083)	771 (737)	679 (572)	86.93 (84.71)	88.30 (87.71)	87.61 (86.18)
	F	156 (166)	27 (21)	45 (40)	394 (386)	3 (3)	625 (616)	394 (386)	231 (230)	148 (125)	63.04 (62.66)	72.69 (75.54)	67.52 (68.50)
	Q	7 (6)	1 (0)	4 (1)	1 (1)	1 (0)	14 (8)	1 (0)	13 (8)	67 (60)	7.14 (0)	1.14 (1.66)	2.44 (0.00)
	Total	74449 (41834)	2792 (2470)	5807 (4655)	542 (511)	68 (60)	83658 (49530)	80379 (46578)	3279 (2952)	3279 (2952)	96.08 (94.04)	96.08 (94.04)	96.08 (94.04)

^†^Classification results for the testing dataset only (24 records from range 200–234) are shown in parenthesis.

**Table 4:** Confusion matrix in setup IIB (personalized scheme) based on datasets in^10^.

Predicted Labels													
		N	S	V	F	Q	Total	TP	FN	FP	*SE* (%)	*PP* (%)	*FS* (%)
Ground Truth	N	39168	935	1295	2767	93	44258	39168	5090	579	88.50	98.54	93.25
	S	311	1328	181	14	3	1837	1328	509	1223	72.29	52.06	60.53
	V	89	275	2628	142	87	3221	2628	593	1587	81.59	62.35	70.68
	F	176	12	109	69	22	388	69	319	2923	17.78	02.31	04.08
	Q	3	1	2	0	1	7	1	6	205	14.28	0.48	0.94
	Total	39747	2551	4215	2992	206	49711	43194	6517	6517	86.89	86.89	86.89

Further, other metrics such as true positives (TP), false positives (FP) and false negatives (FN) parameters are computed for each of the schemes and are summarized in Tables 2, 4 and 3. In setup I scheme, the overall *S*_*E*_, *P*_*P*_, *F*_*s*_ metrics for the 16 classes of ECG signals is computed and presented in Table 2 which is 96.29%, 96.29% and 76.06% respectively. In the personalized scheme i.e. setup IIA following the Ince *et al*.^21^, the overall *S*_*E*_, *P*_*P*_, *F*_*s*_ are 96.08%, 96.08% and 96.08 respectively summarized in Table 3. In setup IIB following the Chazal *et al*.^7^, the overall *S*_*E*_, *P*_*P*_, *F*_*s*_ is 86.89%, 86.89% and 86.89% respectively presented in Table 4.

These results reported under both the setup I, IIA and IIB (i.e. class and personalized schemes) are directly compared with the existing methods reported in the literature and presented in Tables 5 and 6. It can be concluded from Tables 5 and 6, that the proposed method reported higher classification accuracy under both the analysis schemes. In addition, the study classifies more number of ECG signals in the class-oriented scheme. It is evident that the features extracted are significant enough to efficiently represent the input ECG signal in time-frequency space for the developed classifier model, leading to higher accuracy and improved performance achieved by the prototype.

**Table 5:** Comparison table for setup I (class scheme).

Study [Ref.]	Classes	Features	Classifier	Accuracy (%)
Oresko *et al*.^1^	5	RR-interval	NN	90
Cvikl *et al*.^30^	2	RR-interval	OSEA	92.36(*Sp*)
Rodriguez *et al*.^31^	all MIT	Waveform	Decision Tree	96.128
Jeon *et al*.^32^	3	WT	SVM	95.1
**Proposed**	**16**	**DOST**	**LSTSVM-ABC**	**96.29**

^†^NN: Neural Networks, SVM: Support Vector Machine, WT: Wavelet Transform, OSEA: Open Source ECG Analysis Software, ABC: artificial bee colony.

**Table 6:** Comparison table for setup II (personalized scheme).

Study [Ref.]	Classes	Features	Classifier	Accuracy(%)
Hu *et al*.^18^	5	Time-domain	MOE	94.8
De Chazal^7^	5	RR-interval + Morphology	LDA	81.9
Ye^33^	5	WT + ICA + RR	SVM	86.4
**Proposed**	**5**	**DOST**	**LSTSVM-ABC**	**96.08**

^†^LDA: Linear Discriminant Analysis, WT: Wavelet transform, ICA: Independent component analysis, SVM: Support Vector Machines, MOE: Mixture of Experts.

## Discussion

As expected, the performance of developed personalized arrhythmia monitoring platform evaluated under setup II (i.e. personalized scheme) reported worse results than the setup I (i.e class-oriented) scheme, due to inter-individual variability in physiological characteristics between the data of different subjects. The personalized-analysis scheme is suitable for practical applications due to the fact that the training and testing dataset comprises of records of different patients. Further, due to a huge variation in the number of ECG signals among various classes used for training and testing purpose, the performance metrics can be considered as highly significant. The higher accuracy reported under both the schemes i.e. setup I, IIA and setup IIB implicitly signifies the relevance of discriminative features extracted for various categories of heartbeats using discrete orthogonal stockwell transform technique along with the developed classifier model leading to an efficient detection and classification of ECG signals. Further, the prototyping of the proposed method on the hardware platform allows real-time monitoring of ECG signals at the place of patients for direct point-of-care use. The developed hardware platform can be utilized for clinical analysis in hospitals for monitoring different classes (i.e. sixteen classes in class based and five classes in personalized scheme) of arrhythmias with higher accuracy. In this study, the clinical environment scenario is presented by generating real-time signals from the digital data into analog domain for processing and analysis. This kind of implementation provides an assistive diagnostic solution for the cardiovascular diseases which can be utilized by users to lead a health life-style.

This study aims (a) to develop a feature extraction technique to efficiently represent the input ECG signals (b) to propose an efficient classifier for classifying the features into their subsequent categories (c) to evaluate the proposed method for practical applications i.e. in case of personalized assessment scheme (d) hardware implementation of the proposed method to facilitate real-time monitoring of arrhythmias. All these aims on together provide automatic, efficient, universal, fast and reliable solution for general population. The proposed solution have the following advantages such as: (1) one lead used (2) evaluation of the proposed methodology under two schemes i.e. classification of 16 classes and 5 classes in category and personalized scheme respectively (3) high accuracy and sensitivity, (4) lower classification time and (5) more number of classes can be classified using the proposed method.

### Cardiac Summary Report and Storage

The classes of arrhythmias detected by the platform under both the schemes (i.e. setup I and setup II) are stored in a text (‘.txt’) file to generate a daily cardiac summary report regarding the status of heart. The report provides the total number of ECG signals detected by the prototype, along with the number of ECG signals detected in each class. A 1 Gb memory SD card is provided to store the cardiac report summary report, thus allowing off-line analysis by an cardiology expert. In the off-line analysis, the expert can analyze the ECG data (i.e. in digital form) and their classes detected by the platform, thus allowing the reduction in time consumption required for providing preventive measures to the patients. It is to note that a 1 GB card can store the ECG data for 40 days^1^ while the length of recording depend on the memory size of SD card used. Further, during the detection of an arrhythmia by the platform, an alarm is triggered which is a pop-up message warning notification to alert the user. This allows the user to do not continuously monitor the cardiac summary reports and provides a no emergency condition by detecting an arrhythmia at a earlier stage to prevent the users from serious ailments for cardiovascular diseases. The developed prototype can be fabricated to develop a wearable or handheld device for point-of-care use providing real-time feedback regarding the condition of heart to the end users.

## Methods

**Figure 2:**
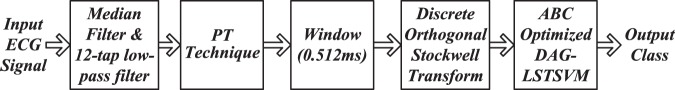
Block diagram of the proposed method.

In this study, a novel ECG signal recognition scheme, i.e. discrete orthogonal stockwell transform (DOST) based artificial bee colony optimized twin least-square support vector machine (ABC-LSTSVM) is explored for automated recognition of cardiac arrhythmias. The block diagram representation of the proposed method is depicted in Fig. 2. Further, the proposed method is implemented on the microcontroller platform to monitor the different categories of ECG signals validated on the benchmark MIT-BIH arrhythmia database.

### MIT-BIH Database

The benchmark MIT-BIH database is chosen to provide the real clinical situation. The database comprises of 48 different subjects. The data are band-pass filtered at 0.1 H–100 Hz and sampled with a rate of 360 Hz which is acquired with 11-bit resolution over 10 mV range. Further, the data acquired is bandpass filtered at 0.1–100 Hz while the database comprises a total of 110109 beat labels. The database also provides the class annotations. In this study, modified limb lead II (MLII) signals are used for the validation while their class annotations are used as ground truth to formulate the results. It is to note that the all the 48 records of the database are utilized to evaluate the proposed method.

### Pre-processing

There are several kinds of noise associated with the raw ECG signal. Among them include the artifacts due to muscle contraction, power-line interference, baseline wander. Hence, pre-processing is essential for an efficient fiducial point detection and recognition of ECG signals. The pre-processing is applied to the noisy ECG signals to improve the signal-to-noise ratio (SNR) and remove the noise. Initially, to remove the baseline wander, the ECG signals are passed through two median filters. The first median filter of 200 ms removes the QRS complex and P wave while the second median filter of 600 ms removes the T wave within the ECG signal. The output signal of the second filter contains the baseline associated with the ECG signal and hence, it is subtracted from the original signal to generate baseline corrected signal. A 12-tap FIR low-pass filter with a cut-off frequency of 35 Hz with equal ripple in the pass and stop bands is used here to remove the high-frequency noise and power-line interference. Finally, the filtered ECG signal is used for processing and analysis.

### R-peak detection and ECG Segmentation

In the practical scenario, the automatic detection of R-peak is essential to evaluate the proposed method entirely for ECG signal analysis. For this purpose, a well established Pan-Tompkins (PT) method^23^ is adopted for the subsequent detection of R-peak in the corresponding ECG signals. The PT method has reported good performance in noisy conditions with less complexity achieving a sensitivity of 99.87% than^24,25^. Therefore, the method is used as a ready-made solution for the R-peak detection in the consecutive ECG signals.

A window of length 0.512 ms is taken across each R-peak to determine the size of each ECG signal. Here, each ECG signal consists of 256 samples i.e. 110 samples before and 145 samples after detected R-peak of the heartbeat. The R-peak detection stage is followed by the feature extraction and classification stages for an efficient recognition of heartbeats.

### Feature extraction using DOST

**Figure 3:**
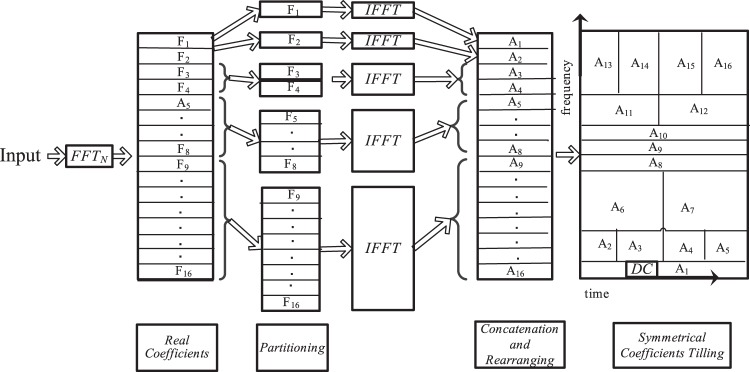
Dataflow of proposed DOST.

The application of discrete orthogonal stockwell^9,26^ transform yields time-frequency morphological coefficients for the different classes of heartbeats. The steps involved in the computation of DOST coefficients are (i) a *N*-point FFT is applied to compute the fourier spectrum of the *N*-length ECG signal *z*(*n*). (ii) a rectangular window function *V*[*p*] = Π_[−*β*/2, *β*/2−1]_(*n*) is multiplied with *z*[*n* + *p*] (iii) A *β*-point inverse FFT is applied to *V*[*p*]*z*[*n* + *p*], for each central frequency *m* = 0, 1, 3, …, 3*β*/2,… to compute the DST coefficients where *p* = 0, *L*/*β*, 2*L*/*β*, …, (*β* − 1)*L*/*β* ensuring that the decomposition is orthogonal. In the inverse-DOST case, a *β*-point FFT is applied to *s*[*q*,*p*] with respect to time index *q* to obtain the windowed fourier spectrum *V*[*p*]*z*[*n* + *p*] for each central frequency *m* = 0, 1, 3, …, 3*β*/2,…. Here, *s*[*q*, *p*] is the DOST coefficient corresponds to the point <q, p>. Note that *V*[*p*] = 1, for *nε*[−*β*/2, …, −*β*/2 − 1] that returns *z*[*p*], *p* = *β*, *β* + 1, …, 2*β*−1, *β* fourier coefficients of the signal. A *N*-point inverse FFT to *z*[*n*] is applied to recover the original signal *z*[*l*]. The total time complexity of DOST algorithm is of the order of Θ(*NlogN* + *NlogN* + *N*). The data flow of the DOST algorithm is depicted in Fig. 3. The time-frequency coefficient vectors are extracted from the corresponding heartbeats that are used as final feature set to recognize the heartbeats into different classes using the ABC-LSTSVM classifier model.

**Figure 4:**
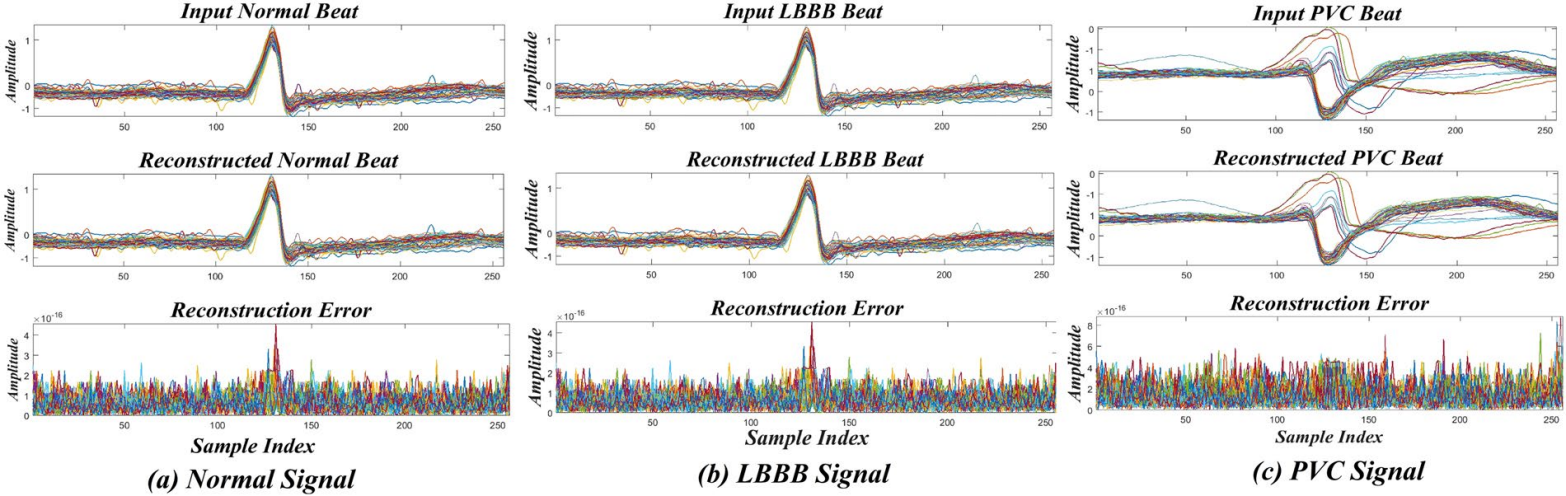
Input, reconstructed signal and error for (**a**) Normal (**b**) LBBB (**c**) PVC signal using DOST.

Figure 4 shows the reconstruction of the normal and the abnormal i.e. LBBB and PVC signals. Here, the error in reconstruction for all the three classes is of the order of 10^−15^. The DOST features of vector of size *N*×1 are extracted for different categories of heartbeats. Therefore, it can be concluded from Fig. 4 that DOST coefficients exhibits better performance while reconstructing the ECG signals. Therefore, the DOST can be considered as a potential tool for extracting significant features from the corresponding ECG signals.

### Feature classification using ABC Optimized least-square twin SVM

An optimal artificial bee colony optimized least-square twin SVM (ABC-LSTSVM) classifier model is developed to classify the DOST features into various categories under the two schemes. In this study, the radial basis function (RBF) kernel is adopted to perform non-linear analysis of the input data by mapping them into the high-dimensional feature spaces. In the recognition phase, directed acyclic graph (DAG) technique^27^ is adopted to provide the multi-class solution to the classifier model. The DAG approach is suitable for practical applications due to the fact that its computational complexity is $O\frac{(P-1){l}^{3}}{{P}^{2}}$, (where *P* is the number of class and *l* is the length of input vector) which is significantly less than the conventional SVM i.e. $\frac{4(K-1){l}^{3}}{{K}^{2}}$. The cost function of both the two hyperplanes are considered as equal i.e. *c*_1_ = *c*_2_ = *C* for significant reduction in computational load of selecting the parameters. The testing time involved is less than those of one-against-one and one-versus-all approaches^27^. In the training phase of DAG-SVM, *N*(*N* − 1) non-parallel hyper-planes and binary classifiers are constructed for a total number of *N* classes. While in testing phase, a binary rooted DAG is used consisting *N*(*N* − 1)/2 internal decision nodes (binary classifiers) and *N* leaves. The searching range of penalty *C* and kernel argument *γ* parameters are in the range of [2^−5^, 2^10^] and [2^−10^, 2^5^] respectively. The artificial bee colony (ABC)^28^ technique is employed to search the optimal learning parameters for the least-square twin support vector machine classifier.

**Figure 5:**
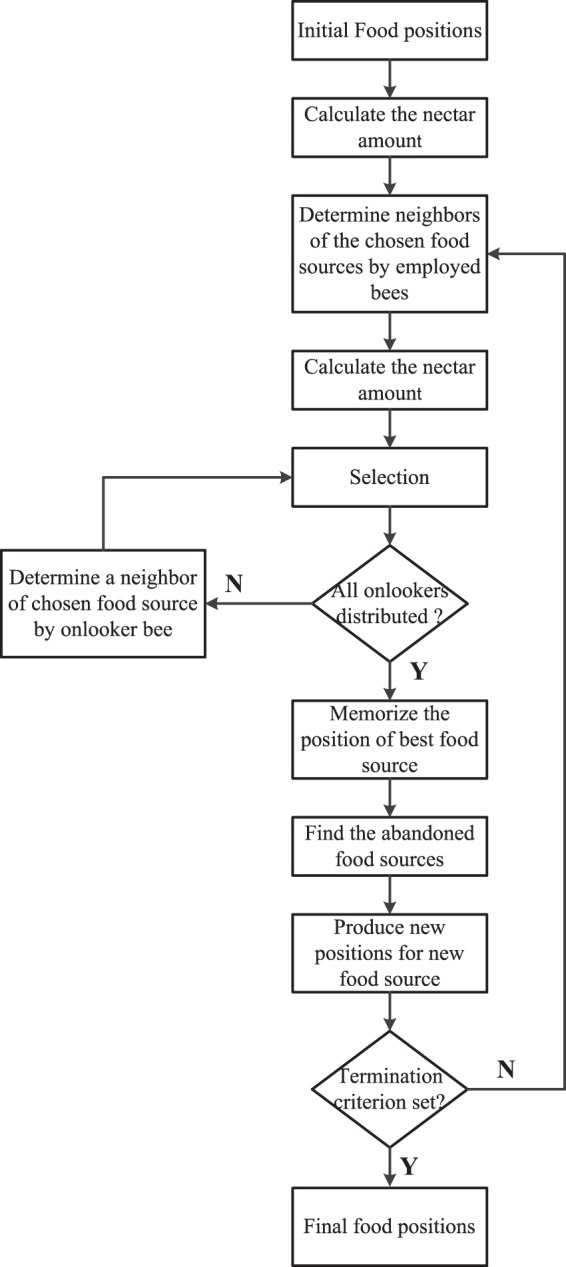
Flowchart of ABC technique.

The ABC aims to gradually optimizes the least-square twin SVM classifier by a) employing the best features to distinguish between different categories of heartbeats automatically and b) selecting best learning parameters for the classification model. The flowchart of the ABC technique is presented in Fig. 5 and its pseudo code is summarized here.

**Figure:**
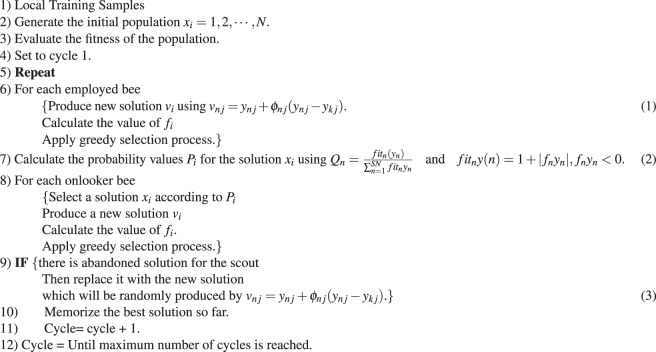
**Pseudo code of ABC algorithm**.

In the ABC algorithm, the parameters considered for performing the experiments are summarized in Table 7. ABC evaluates the fitness of each food source at each iteration and determines the optimal network. In the training phase, the classifier parameters i.e *C* and *γ* are gradually tuned using the ABC algorithm following the m-fold cross-validation strategy^29^ where the simple SV count is employed as a fitness criteria. The ten-fold cross-validation is performed on the training as well as testing dataset i.e. entire dataset using the optimal parameter values of *C* and *γ* to achieve better accuracy. It is to note that the whole procedure is performed for the datasets under both the analysis schemes presented in section 0 i.e. for both the schemes different values of *C* and *γ* are obtained. During testing phase, the testing dataset is utilized for estimating the classification accuracy for ECG signal class which is reported in terms of confusion matrix.

**Table 7:** Parameters in the ABC Technique.

Parameters In the ABC Technique	
Number of Bees (Onlooker + Employed Bees)	200 (50 + 150)
Maximum Number of cycles (MCN)	500
No. of Iterations for Onlooker Bees	200
No. of Food sources	25

### Hardware prototyping and implementation of proposed method

This study aims to develop microcontroller-based platform technology having the capability to perform real-time ECG reception, extraction of features, and heartbeat recognition. To demonstrate the feasibility of the idea, a proof-of-concept prototype is developed using commercially available ARM9 platform in the laboratory experimental setup.

#### Laboratory prototype

**Figure 6:**
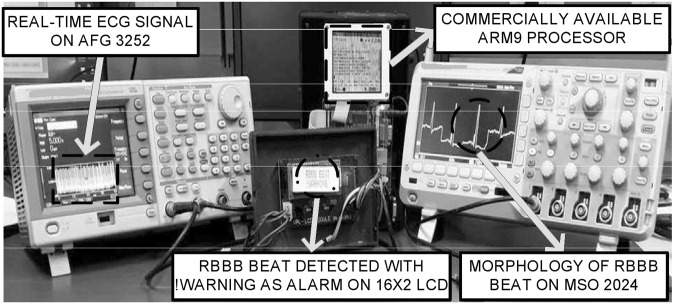
Experimental laboratory setup.

The proposed method is implemented on the ARM based hardware test platform in real scenario. The experiments are performed to evaluate the proposed method and monitor the different classes of ECG signals. The #C++ programming language is used to develop the proposed method while the debugging environment is linux to program the ARM processor. The gcc compiler compiles the program to generate the output file. While the output file of the program is executed, the processor platform starts predicting the ECG signals. The real-time ECG signals are generated using the arbitrary function generator (AFG 3252) while their class detected by the platform is observed on the liquid crystal display (i.e. 16 × 2) interfaced with the processor platform. The AFG 3252 provides two-channel analog output system from which these real-time signals using channel I are taken through BNC crocodile connection cable (i.e. with an impedance of 50 Ω) to the platform as input for further processing and analysis. In addition, the morphology of the signals can be seen on mixed signal oscilloscope (i.e. MSO 2024B). Figure 6 depicts the laboratory experimental setup for the developed hardware test platform.

### Implementation of the proposed method

Initially, the training of the hardware test platform is performed in off-line mode. In off-line mode, all the parameters are computed using MATLAB software package [R2014a; Version 7.14.0.739 installed on PC]. Once the model has been determined, the training of the model is performed and the parameters such as support vectors (*SVs*), alphas (*α*_*i*_) and variables are stored in the memory of the hardware test platform through a serial port. The experiments performed on the datasets under both the schemes i.e. case I and case II will yield different values of *C* and *γ*. The platform is evaluated on the testing dataset signals to estimate the performance.

**Figure 7:**
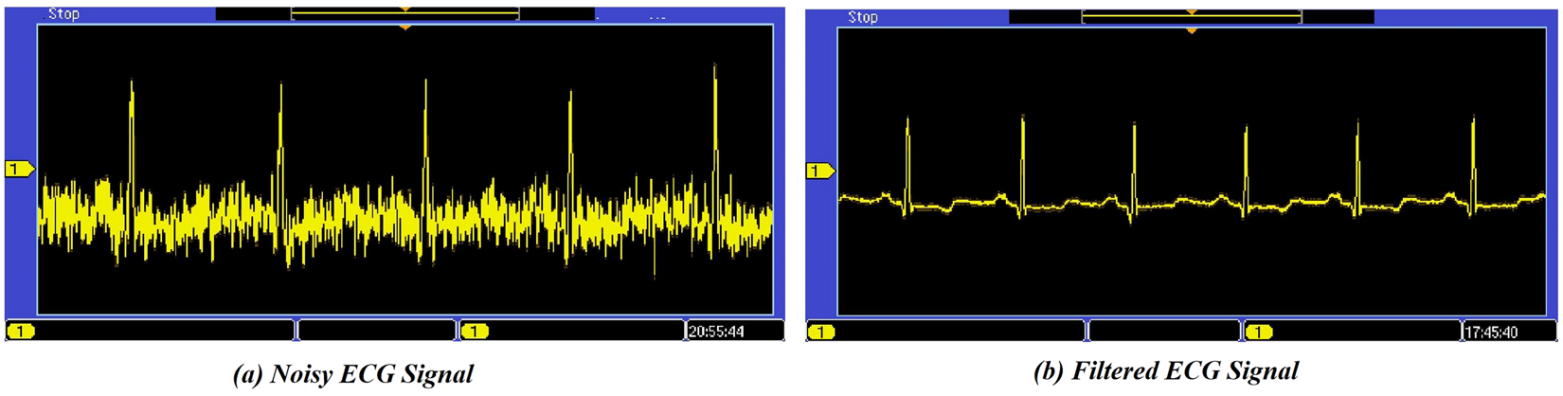
Noisy ECG signal and its pre-processing.

**Figure 8:**
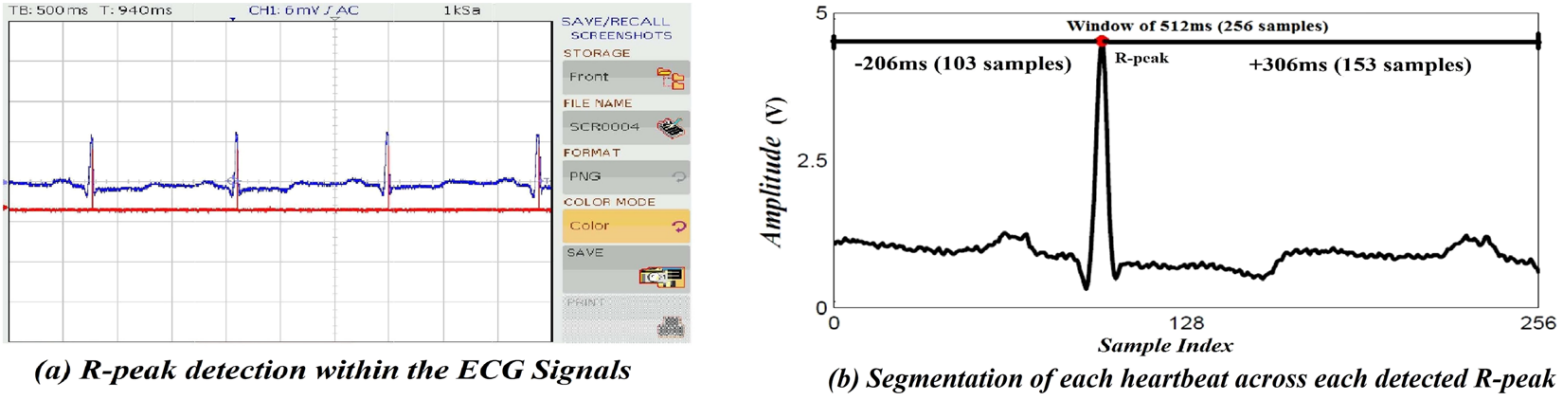
R-peak detection and ECG segmentation.

The ECG signals from the testing dataset are randomized, re-annotated and generated in a text file which is transferred to the arbitrary function generator (i.e. AFG 3252) for real-time generation of input signals. In the MIT-BIH arrhythmia database, the ECG data available have a maximum dynamic range of ±5 mV. The data is converted in the range of 0–5 V using the AFG for its processing by a typical 10-bit analog-to-digital (ADC) converter. The sampling rate of the ADC is set to 500 Hz. Further, a pause is added; so that the packages should arrive at the same sampling rate as the ADC. The data is encoded with a fixed point math by using 32-bit representation. Thenafter, the proposed method is applied for processing the acquired input signal. The raw ECG signals are preprocessed to remove noise and improve the SNR of the ECG signals. In Fig. 7, shows the two channel mixed signal oscilloscope (MSO) in which the noisy along with the filtered ECG signals are observed in channels I and II respectively. Further, the R-peak of the filtered ECG is detected using the PT algorithm^23^ which is depicted in Fig. 8(a). Figure 8(a), shows the R-peak detection in the consecutive ECG signals on the digital signal oscilloscope. The R-peak detection is followed by a window of length 256 samples, i.e. 0.712 ms chosen across each R-peak to determine the length of ECG segments and depicted in Fig. 8(b).

Further, the ECG segments are passed through the feature extraction and classification stages. In the feature extraction stage, the time-frequency features are extracted. The twin SVM model is developed and exploited in off-line mode for training purpose. The performance of SVM classifier is presented at convergence during the optimization procedure using ABC. The real-time testing is performed on the testing dataset where once the features reaches this stage, the computation gets started and the input ECG signals are recognized by the test platform. During testing, the category of ECG signals detected is displayed on the liquid crystal display (i.e. 16 × 2 LCD) module while their morphology is displayed on mixed signal oscilloscope (i.e. MSO 2024B) in the experimental laboratory setup depicted in Fig. 6. In Fig. 6, a right bundle branch block (RBBB) signal is detected at that instant of time and consequently, the other classes of the ECG signals are detected by the developed platform.

## Conclusions

This study presents a novel method i.e. discrete orthogonal stockwell transform for feature extraction and artificial bee colony (ABC) optimized least-square support vector machines for the classification of the features extracted for each of the ECG signals into their subsequent categories. The proposed method is implemented on ARM processor platform, validated on the MIT-BIH arrhythmia database and evaluated under two schemes i.e. class and personalized schemes to monitor the different classes of arrhythmias. The evaluation under personalized scheme is suitable for practical scenario. A higher accuracy of 96.29% and 86.89% is reported under class and personalized schemes respectively than the existing works in the literature. The platform can be utilized by end users to maintain a daily record of the cardiac health status to enhance the healthcare for cardiovascular diseases and lead a healthy life style. Further, the platform can also be utilized in hospitals to analyze the long-term ECG recordings, thus reducing the time of cardiologist in providing the necessary preventive measures to the patients.

## References

[1] Oresko J (2010). A Wearable Smartphone-Based Platform for Real-Time Cardiovascular Disease Detection Via Electrocardiogram Processing. IEEE Trans. Inf. Technol. Biomed..

[2] Garcia G, Moreira G, Menotti D, Luz E (2017). Inter-Patient ECG Heartbeat Classification with Temporal VCG Optimized by PSO. Sci. Rep. (Nature).

[3] Kiranyaz S, Ince T, Gabbouj M (2017). Personalized Monitoring and Advance Warning System for Cardiac Arrhythmias. Sci. Rep. (Nature).

[4] Li H, Yuan D, Ma X, Cui D, Cao L (2017). Genetic algorithm for the optimization of features and neural networks in ECG signals classification. Sci. Rep. (Nature).

[5] Maršánová L (2017). ECG features and methods for automatic classification of ventricular premature and ischemic heartbeats: A comprehensive experimental study. Sci. Rep. (Nature).

[6] Qin Q, Li J, Zhang L, Yue Y, Liu C (2017). Combining Low-dimensional Wavelet Features and Support Vector Machine for Arrhythmia Beat Classification. Sci. Rep. (Nature).

[7] Chazal PD, Dwyer MO, Reilly RB (2004). Automatic Classification of Heartbeats Using ECG Morphology and Heartbeat Interval Features. IEEE Trans. Biomed. Eng..

[8] Minami K, Nakajima H, Toyoshima T (1999). Real-time discrimination of ventricular tachyarrhythmia with fourier-transform neural network. IEEE Trans. Biomed. Eng..

[9] Raj S, Ray KC, Shankar O (2016). Cardiac arrhythmia beat classification using dost and pso tuned svm. Comput. Methods Programs Biomed..

[10] Raj S, Ray KC (2017). Ecg signal analysis using dct-based dost and pso optimized svm. IEEE Trans. Instrum. Meas..

[11] Raj S, Chand GSSP, Ray KC (2015). Arm-based arrhythmia beat monitoring system. MICPRO. (Elsevier).

[12] Wang Y, Doleschel S, Wunderlich R, Heinen S (2016). Evaluation of Digital Compressed Sensing for Real-Time Wireless ECG System with Bluetooth low Energy. J. Med. Syst..

[13] Wang Y, Doleschel S, Wunderlich R, Heinen S (2015). A Wearable Wireless ECG Monitoring System With Dynamic Transmission Power Control for Long-Term Homecare. J Med Syst.

[14] Llamedo M, Martinez JP (2012). An automatic patient-adapted ECG heartbeat classifier allowing expert assistance. IEEE Trans. Biomed. Eng..

[15] Raj S, Luthra S, Ray KC (2015). Development of handheld cardiac event monitoring system. 13th IFAC and IEEE Conf. Programmable Devices and Embedded Systems (PDES).

[16] Raj S, Ray KC (2018). Sparse representation of ECG signals for automated recognition of cardiac arrhythmias. Expert Syst. Appl..

[17] Raj, S. & Ray, K. C. Application of variational mode decomposition and ABC optimized DAG-SVM in arrhythmia analysis. 17th Int. Symp. Embedded Computing and System Design (ISED), 1–5 (2017).

[18] Hu Y (1997). A patient-adaptable ECG beat classifier using a mixture of experts approach. IEEE Trans. Biomed. Eng..

[19] Moody GB, Mark RG (2001). The impact of mit-bih arrhythmia database. IEEE Trans. Biomed. Eng..

[20] Testing and Reporting Performance Results of Cardiac Rhythm and ST Segment Measurement Algorithms, ANSI/AAMI EC57:1998 standard, Association for the Advancement of Medical Instrumentation (1998).

[21] Ince T, Kiranyaz S, Gabbouj M (2009). A Generic and Robust System for Automated Patient-specific Classification of Electrocardiogram Signals. IEEE Trans. Biomed. Eng..

[22] Kiranyaz S, Ince T, Gabbouj M (2016). Real-Time Patient-Specific ECG Classification by 1D Convolutional Neural Networks. IEEE Trans. Biomed. Eng..

[23] Pan J, Tompkins WJ (1985). A Real-Time QRS Detection Algorithm. IEEE Trans. Biomed. Eng..

[24] Li H, Wang X (2013). Detection of electrocardiogram characteristic points using lifting wavelet transform and Hilbert transform. T I. Meas Control..

[25] Li H, Wang X, Chen L, Li E (2014). Denoising and R-Peak Detection of Electrocardiogram Signal Based on EMD and Improved Approximate Envelope. Circ Syst Signal Pr..

[26] Stockwell RG (2007). A basis for efficient representation of the s-transform. Elsevier-Dig. Sig. Pro..

[27] Tomar D, Agarwal S (2015). A comparison on multi-class classification methods based on least squares twin support vector machine. Knowledge Based Systems.

[28] Karaboga, D. An idea based on honey bee swarm for numerical optimization, Technical Reports TR06, Oct. (2005).

[29] Stone M (1974). Cross-validatory choice and assessment of statistical predictions. J. Roy. Statist. Soc. B.

[30] Cvikl M, Zemva A (2010). Fpga-oriented hw/sw implementation of ecg beat detection and classification algorithm. Dig. Sig. Pro. (Elsevier).

[31] Rodriguez J, Goni A, Illarramendi A (2005). Real-time classification of ECGs on a PDA. IEEE Trans. Inf. Tech. in Biomed..

[32] Jeon T, Kim B, Jeon M, Lee B (2014). Implementation of a portable device for real-time ECG signal analysis. BioMedical Engineering OnLine.

[33] Ye C, Kumar B, Coimbra M (2012). Heartbeat classification using morphological and dynamic features of ecg signals. IEEE Trans. Biomed. Eng..

